# Recent cannabis use affects the association between baseline immune markers and long-term outcomes in psychosis

**DOI:** 10.1038/s41398-025-03498-x

**Published:** 2025-08-14

**Authors:** Isabel Kreis, Kristin Fjelnseth Wold, Gina Åsbø, Camilla Bärthel Flaaten, Magnus Johan Engen, Siv Hege Lyngstad, Line Hustad Widing, Mashhood Ahmed Sheikh, Maren Caroline Frogner Werner, Eivind Bakken, Thor Ueland, Nils Eiel Steen, Ingrid Melle

**Affiliations:** 1 Centre for Precision Psychiatry, Institute of Clinical Medicine, Faculty of Medicine, University of Oslo, Oslo, Norway; 2 Section for Clinical Psychosis Research, Department of Research and Innovation, Division of Mental Health and Addiction, Oslo University Hospital, Oslo, Norway; 3 Institute of Clinical Medicine, University of Oslo, Oslo, Norway; 4 Department of Psychology, Faculty of Social Sciences, University of Oslo, Oslo, Norway; 5 Nydalen District Psychiatric Center, Division of Mental Health and Addiction, Oslo University Hospital, Oslo, Norway; 6 Department of Child and Adolescent Psychiatry, Division of Mental Health and Substance Use, Diakonhjemmet Hospital, Oslo, Norway; 7 Research Institute of Internal Medicine, Oslo University Hospital, Rikshospitalet, Oslo, Norway; 8 Division of Clinical Neuroscience, Department of Research and Innovation, Oslo University Hospital, Oslo, Norway; 9 Section for Precision Psychiatry, Department of Research and Innovation, Division of Mental Health and Addiction, Oslo University Hospital, Oslo, Norway; 10 Thrombosis Research Center (TREC), Division of Internal Medicine, University Hospital of North Norway, Tromsø, Norway; 11 Department of Psychiatric Research, Division of Mental Health and Substance abuse, Diakonhjemmet Hospital, Oslo, Norway; 12 Adult Psychiatry Department, Institute of Clinical Medicine, Faculty of Medicine, University of Oslo, Oslo, Norway

**Keywords:** Prognostic markers, Schizophrenia

## Abstract

Aberrant levels of blood markers reflecting inflammation and immune system activation have been implicated in psychotic disorders and linked to psychotic symptom severity. However, their predictive value for the long-term course of psychotic symptoms as well as the potential confounding and moderating role of cannabis use remain underexplored. We tested if baseline levels of immune markers previously linked to psychotic symptoms or treatment response (CRP, IL-1RA, sIL-2R, sTNFR1, sgp130) predicted 10-year outcomes in a first-episode psychosis sample (N = 320), and whether associations were moderated by baseline cannabis use. We assessed psychiatric (re)admissions and number of psychotic episodes during each year of the follow-up period, as well as change in positive psychotic symptom severity from baseline. Apart from sTNFR1, none of the immune markers significantly predicted psychosis outcomes independently of cannabis use. Baseline sTNFR1 was linked to lower risk of both (re)admissions and psychotic episodes, with an increasingly negative association over time. The statistical effects of CRP, IL-1RA, and sgp130 were all dependent on cannabis use. Specifically, negative (CRP, IL-1RA) or positive associations (sgp130) with psychiatric (re)admission risk or psychotic episode risk were observed in cannabis users only. Similarly, sgp130 was negatively associated with symptom change in cannabis users only. Some of these associations varied by follow-up year of the measured outcome (sgp130, IL-1RA). These findings challenge the prognostic and etiological significance of baseline immune markers for the course of positive psychotic symptoms and emphasize the importance of accounting for cannabis use.

## Introduction

Positive psychotic symptoms, such as delusions and hallucinations, are the transdiagnostic defining symptoms of psychotic disorders, but their long-term course is highly heterogeneous [1, 2]. Hence, prediction of related outcomes, such as risk of relapse, psychiatric hospitalization, or treatment resistance, remains challenging [1, 3–5], prompting a search for biomarkers that may improve existing prediction models [5–7].

Candidate biomarkers include measures of inflammatory processes and immune system activation, as growing evidence implicates their dysregulation in psychotic disorders such as schizophrenia [8–10]. This dysregulation is often reflected in aberrant circulating levels of immune markers, such as pro- and anti-inflammatory cytokines and their receptors as well as robust down-stream markers reflecting overall systemic inflammation such as C-reactive protein (CRP) [8, 9, 11]. While some of these aberrancies seem to be state-dependent, with aberrant levels mainly observed in acute but not chronic illness, some immune markers are considered ‘trait’ markers, with aberrant levels seen in acute and chronic illness phases [11, 12]. Such trait markers include both markers with primarily pro-inflammatory [CRP, interleukin (IL)-1β, IL-6, IL-8, tumor necrosis factor (TNF)-α] and anti-inflammatory [IL-1RA, IL-10] effects, or both [soluble interleukin-2 receptor (sIL-2R)], all circulating at elevated levels in both acute and chronic schizophrenia [12]. Elevated levels of anti-inflammatory markers may reflect a compensatory response to low-grade inflammation in psychosis [12, 13].

Immune marker aberrancies are not restricted to schizophrenia but also present in other severe mental disorders, including bipolar disorder and diagnostically heterogeneous samples of FEP [8, 14–19]. Furthermore, circulating immune marker levels have been linked to clinical indicators of illness severity, positive psychotic symptoms and number of relapses, treatment resistance, as well as general psychopathology [9, 20–28]. Hence, these markers might prove helpful in predicting the long-term course of psychotic symptoms and recurrence patterns of psychotic episodes. However, longitudinal studies are lacking and even fewer have considered FEP samples and long-term outcomes past the first year. Furthermore, findings have been inconsistent, particularly concerning outcomes capturing positive psychotic symptomatology: Concerning short-medium term outcomes (~1 year) in multi-episode schizophrenia patients, baseline immune markers were predictive of positive symptom change in one [29] but not another study [30], and not linked to relapse risk [31]. In some FEP samples, baseline immune markers, including CRP, IL-6, and soluble TNF-α receptor 1 (sTNFR1; reflective of TNF-α activity), predicted treatment response and positive psychotic change several months [32, 33] or one year later [34], while other studies found no association between baseline immune markers and short- [35] or medium-long term psychiatric outcomes (need for specialist care/psychiatric readmission) [36, 37].

These inconsistent findings could be related to differences in methodology and samples, including assessment of confounding or moderating factors. In the context of positive psychotic symptoms, relapse risk, and treatment resistance, a candidate confounder or moderator of relevance is cannabis use. Cannabis use has been linked to worsening of positive psychotic symptoms, increasing the risk for psychotic relapse and rehospitalization [38–40]. Moreover, it can modulate immune system activity [41, 42].

Immune cells express two of the main receptors of the endocannabinoid system, CB2 and (to a lesser extent) CB1, through which both endogenous and exogenous cannabinoids exert their effects [42–44]. Both endogenous and exogenous cannabinoids can influence leucocyte proliferation, T-cell and macrophage apoptosis, as well as cytokine secretion from these cells [41, 42]. In vitro and in vivo studies suggest a primarily immunosuppressive and anti-inflammatory effect of cannabinoids [41, 42, 45], including the exogenous cannabinoids Δ9-tetrahydrocannabinol (Δ9-THC) and cannabidiol (CBD), the most researched phytocannabinoids found in the Cannabis sativa plant. In humans, lifetime [46, 47], recent [48, 49], and heavy current [50] cannabis use has been associated with lower levels of various immune markers, including CRP, IL-6, TNF, and soluble TNF-α receptor 2 (sTNFR2).

Very few studies have investigated the relationship between immune markers and cannabis use in psychosis. Here, lower levels of pro-inflammatory and higher levels of anti-inflammatory markers were observed in individuals testing positive for cannabis via urinary drug screen [51–54] or reporting current [19] or recent [55] cannabis use, than in individuals who did not, and cannabis use cessation was found to increase CRP levels while improving clinical symptoms after four weeks [51, 52]. Considering the immunosuppressant and detrimental clinical effects of cannabis, its use is a highly relevant potential confounder of the association between immune marker levels and the course of positive psychotic symptoms. Furthermore, cross-sectional studies suggest an additional moderating (or ‘quasi-moderating’ [56]) role of cannabis use, with daily and adolescent cannabis use interacting with immune status to predict psychosis risk [14] and associations between cannabis use and psychotic symptom severity in established psychosis depending on current cannabis use status [53, 54]. However, to our knowledge, the confounding and possibly moderating role of cannabis use has so far not been adequately addressed in longitudinal studies on the relationship between immune markers and the long-term course of positive psychotic symptoms.

Our study aims to address the lack of studies on the long-term predictive potential of immune markers and on the putative role of cannabis use in moderating immune marker associations with psychosis outcomes. In a FEP sample, we measured multiple immune markers at baseline, reflecting activity in immunoinflammatory pathways that a) are assumed to be altered in a state-independent manner in psychotic disorders [12], and b) have previously been linked to treatment resistance, illness severity, and/or positive symptoms across different psychotic disorders in cross-sectional studies (CRP: e.g., [22–24, 57–59]; IL-1RA: e.g., [25, 60, 61]; sTNFR1: e.g., [26, 60, 62, 63]; sIL-2R: e.g., [28, 64, 65]; IL-6 pathway: e.g., [23, 24, 27, 59, 66]). Associations between these markers and the 10-year course of positive psychotic symptoms were investigated, captured by psychiatric (re)admissions, a commonly used indicator of relapse [67, 68], as well as by self-reports of concrete psychotic episodes and change in positive symptom severity from baseline to 10-year follow-up. Recent cannabis use at baseline was controlled for in analyses, and its possibly moderating role was investigated with interaction terms.

## Methods

### Participants

The study sample included patients recruited to the Thematically Organized Psychosis (TOP) project during their first adequate treatment from Oslo and two adjacent counties (Østfold and Innlandet) in Norway between 2004 and 2012. All were diagnosed with a non-affective psychotic disorder or a bipolar disorder with psychotic symptoms [69].

Of the initial 513 individuals with FEP, only those with plasma levels of at least one immune marker of interest, measured within ± 6 months of clinical baseline interviews, were selected for the current study (see below: *Immune markers*). Participants with CRP values ≥ 10 mg/L were excluded to rule out the influence of acute infections. This resulted in a baseline sample of *N* = 320 individuals (Supplementary Figure S1). Baseline data of this sample was linked to the National Patient Registry (NPR) to obtain information about contacts with specialized psychiatric health services within a 10-year follow-up time frame. For a subsample of these 320 individuals (*n* = 132), personal interview data collected after approximately 10 years (*M* = 9.30, *SD* = 1.56) was available for at least one of the measures of interest described in detail below: psychotic episodes during follow-up (*n* = 126) and change in positive psychotic symptom severity from baseline to follow-up (*n* = 130).

The study and all contributing procedures were conducted in line with the Declaration of Helsinki and approved by the Regional Committee for Medical Research Ethics South East Norway. All participants provided written informed consent before participation, which included permission to link their study data with data obtained from the NPR.

### Clinical and cannabis use assessments

At baseline, psychiatric diagnoses were confirmed with the Structured Clinical Interview for Mental Disorders (SCID-I) [70], according to the Diagnostic and Statistical Manual of Mental Disorders (DSM-IV). Demographic and clinical information was recorded, including age, sex, consumption of tobacco and/or nicotine products (daily nicotine yes vs. no), and current (antipsychotic) medication. To represent antipsychotic medication load, the daily dose of the currently prescribed primary antipsychotic (PDD) was divided by the corresponding Defined Daily Dose [71] (PDD/DDD ratio).

Recent cannabis use at baseline was measured as self-reported use during the past 6 months in a dichotomized manner (yes vs. no). Since the focus of the current study was to assess cannabis use status as a potential confounder and/or moderator as opposed to its predictive value of psychosis outcomes per se, and considering its comparatively acute effects on immune markers, lifetime use or frequency of use were not of primary interest. The dichotomized operationalization of recent or current use in this context is also more in line with previous, mainly cross-sectional, research in this field [19, 51–55].

At both baseline and 10-year follow-up, symptom severity was assessed with the Positive and Negative Symptom Scale (PANSS) [72]. Symptoms were grouped according to the 5-factor model proposed by Wallwork et al. [73], a recommended model for assessing symptoms in FEP [74]. Only the positive symptoms subscale was selected for the current study, consisting of the average rating across items p1 (delusions), p3 (hallucinations), p5 (grandiosity), g9 (unusual thought content), with larger scores reflecting higher symptom severity. Change in positive psychotic symptom severity from baseline to follow-up was calculated as ΔPANSS-Pos = PANSS-Pos baseline – PANSS-Pos follow-up. Here, larger positive scores indicate more improvement, i.e., a larger decrease in symptom severity, while negative scores indicate deterioration. Only participants with available ΔPANSS-pos scores were included in PANSS-based analyses (*n* = 130).

At follow-up, the number of episodes with manifest psychotic symptoms lasting ≥1 week during each year of the 10-year follow-up period was assessed retrospectively, using a time chart with a visual analog timeline. For analyses, these data were dichotomized per year (psychotic episode yes vs. no), and only participants with information on at least one of the 10 years were included (*n* = 126; average number of missing years: *M* = 0.85, *SD* = 1.50).

### Registry data

Data from the NPR encompassed dates, duration, and level of care (inpatient care, outpatient contacts, or day treatment) of any contacts with specialized health services, as well as the primary ICD-10 diagnoses associated with a given contact. For each participant of the baseline sample (*N* = 320), any contacts due to schizophrenia spectrum diagnoses (ICD-10 codes F20-F29) or bipolar disorder (F31) that occurred during the ten years following the study baseline were extracted per year. For analyses, these data were dichotomized per year to reflect psychiatric (re)admission (yes vs. no). Since the digitally available data from the NPR only covered a time frame from 01.01.2008 to 31.12.2020, data were missing for some of the earlier years in earlier recruited participants and some of the later years in later recruited participants. For all 320 participants, information was available for at least four of the 10 included follow-up years (average number of missing years: *M* = 0.93, *SD* = 1.18).

### Immune markers

Immune markers of interest measured at baseline included: CRP, a common marker of inflammation and infection, circulating at elevated levels during inflammatory conditions [75]; IL-1RA, a protein reflecting IL-1 concentrations and inhibiting IL-1 signaling, counteracting its pro-inflammatory effects by binding to IL-1 receptors [76]; sIL-2R, a soluble cytokine receptor reflecting T-cell activity and an ongoing immune response, with the potential for both pro- and anti-inflammatory effects through augmentation or inhibition, respectively, of pro-inflammatory IL-2 activity [77]; and sTNFR1, a soluble cytokine receptor reflecting TNF (α and β) activity and inhibiting its pro-inflammatory actions [78, 79]. To gauge activity in the IL-6 signaling pathway, sgp130 was measured, a soluble cytokine receptor with inhibiting effects on primarily the pro-inflammatory IL-6 trans-signaling [80–82]. While previous research on immune markers in psychosis has often focused on IL-6, sgp130 was selected here because, just like the other selected markers, it is assumed to be adequately robust, circulating at reasonably high plasma concentrations even in low-inflammation conditions, and with a sufficiently long biological half-life. Plasma levels of cytokines are not always detectable and/or reproducible [83], especially in low-inflammation conditions and when the respective cytokines are typically circulating at levels close to the detection threshold of conventional assays. Cytokine receptors, however, are often present at higher levels, are therefore more reliably detectable (e.g., sTNFR1 instead of TNF-α, sgp130 instead of IL-6) [60, 84, 85] and were thus selected for this study.

Baseline blood samples were taken approximately three weeks after the baseline clinical assessments. Occasionally, blood sampling was conducted earlier or had to be postponed, and samples measured within ± 6 months of the clinical interviews were still included in current analyses, overlapping with the time window of cannabis use assessments. For n = 11 participants, the time difference between baseline clinical assessments and blood sampling was >90 days. The average absolute time difference between baseline clinical assessments and blood sampling was *M* = 21.72 days (*SD* = 23.95). Baseline blood samples were taken between 8:00 and 17:00 from the antecubital vein and collected in EDTA vials. After overnight storage at 4 °C, plasma was extracted and stored at −80 °C. Immunological analyses of the thawed samples were conducted in 2013 at the Research Institute of Internal Medicine, Oslo University Hospital, with the accumulated storage time depending on the time point of sampling (*M* = 4.89 years, *SD* = 1.97 in the baseline sample, *N* = 320). Plasma levels of the different immune markers were measured in duplicate using enzyme immunoassays (EIA) with antibodies from R&D Systems (Minneapolis, MN, USA) in a 384-well plate, a SELMA (Jena, Germany) pipetting robot and a BioTek (Winooski, VT, USA) dispenser/washer. An Enzyme-Linked Immunosorbent Assay (ELISA) plate reader was used to measure absorbance at 450 nm, with wavelength correction set to 540 nm. Test reproducibility was good, with all intra- and inter-assay coefficients of variation <10%. Concentrations were measured in mg/L (CRP), ng/ml (sIL-2R, sTNFR1, sgp130) and pg/ml (IL-1RA). Values were log- and z-transformed for analyses. CRP concentrations were available for all 320 participants, but for four participants levels of IL-1RA, sTNFR1, sIL-2R, and sgp130 were missing.

### Statistical analyses

Associations between immune marker levels and the different psychosis outcomes were assessed with logistic mixed effects models (psychiatric admissions, psychotic episodes) with a random intercept for participants, and linear regression models (ΔPANSS-Pos), implemented separately for the different immune markers. Predictors included main effects of the respective immune marker, cannabis use, and follow-up year (in mixed models only), as well as interactions between immune marker, cannabis use, and follow-up year (in mixed models only). Follow-up year interactions were included to consider that the predictive value of baseline immune marker levels may change as the illness progresses. All models were adjusted for baseline characteristics (age, sex, daily nicotine consumption, antipsychotic medication). ΔPANSS-Pos regression models were additionally adjusted for PANSS-Pos baseline scores, and scores were log-transformed prior to change score calculation to normalize model residuals. Testing was conducted two-sided, with a significance level of α = 0.05. As an incidence sample from a low-incidence disorder, the sample size is determined by baseline recruitment. Nevertheless, the relatively large sample size, recruited from a large and representative catchment area, and the use of a repeated measures approach, provide a strong foundation for detecting meaningful patterns in the data.

Analyses were implemented in R (version 4.2.3) [86], using the lme4 package for logistic mixed effects models (version 1.1-32) [87] and the sjPlot package for visualization of interaction effects (version 2.8.15) [88]. Model diagnostics were tested by inspection of residual distributions, using the DHARMa package (version 0.4.6) [89] for mixed models. Here, no issues concerning outliers, over- or underdispersion, or non-normality were detected, while minor deviations in quantile plots were tolerated.

## Results

### Study sample

Baseline characteristics of the 320 FEP participants are summarized in Table 1. Contrasts of immune marker levels by cannabis use status are provided in the Supplementary Information but are not of main interest for the current study. No significant differences were observed (see Supplementary Table S1).

**Table 1:** Descriptive statistics of central baseline variables and covariates (N = 320).

	*N*	*%*
Sex (f/m)	134/186	41.9/58.1
Diagnosis		
Bipolar Disorder	78	24.4
Other Psychosis	88	27.5
Schizophrenia Spectrum	154	48.1
Daily nicotine (n/y)^a^	146/171	45.6/53.4
Cannabis use (n/y)^b^	242/71	75.6/22.2
	***Median***	***IQR***
Age	25.00	11.25
PDD/DDD^c^	0.67	0.94
CRP^d^	1.81	2.84
IL-1RA^e,f^	204.50	307.12
sIL-2R^e,g^	0.26	0.18
sgp130^e,g^	205.37	56.09
sTNFR1^e,g^	1.68	0.61

Concentrations of immune markers (CRP, IL-1RA, sIL-2R, sgp130, sTNFR1) are shown on their original, non-transformed scale.

*IQR* interquartile range, *PDD/DDD* antipsychotic medication load.

^a^Missing: n = 3.

^b^Missing: n = 7.

^c^Missing: n = 1.

^d^mg/L

^e^Missing: n = 4.

^f^pg/ml.

^g^ng/ml.

### Registry-based analyses

Logistic mixed effects models with psychiatric admission per follow-up year as the dependent variable revealed no significant main effects of either immune marker levels or cannabis use (Table 2). However, there were significant interactions between immune markers and cannabis use and/or year for the models including either CRP, IL-1RA, sgp130, or sTNFR1 as predictors.

**Table 2:** Main and interaction effects for each immune marker model predicting psychiatric admission (registry-based analyses).

	CRP		IL-1RA		sIL-2R		sgp130		sTNFR1	
*predictor*	*OR [CI]*	*p*	*OR [CI]*	*p*	*OR [CI]*	*p*	*OR [CI]*	*p*	*OR [CI]*	*p*
IM	1.06 [0.68, 1.65]	0.787	0.75 [0.48, 1.17]	0.201	1.11 [0.73, 1.70]	0.622	1.10 [0.64, 1.87]	0.734	1.21 [0.77, 1.90]	0.418
Cannabis	1.08 [0.40, 2.9]	0.881	1.38 [0.50, 3.79]	0.534	1.08 [0.40, 2.90]	0.884	1.04 [0.38, 2.85]	0.938	1.08 [0.40, 2.90]	0.883
Year	0.72 [0.68, 0.75]	<0.001	0.72 [0.68, 0.76]	<0.001	0.72 [0.68, 0.76]	<0.001	0.72 [0.68, 0.75]	<0.001	0.72 [0.68, 0.75]	<0.001
IM × Cannabis	0.27 [0.10, 0.72]	0.009	0.20 [0.07, 0.55]	0.002	0.70 [0.20, 2.46]	0.573	0.50 [0.22, 1.12]	0.091	0.65 [0.27, 1.53]	0.318
IM × Year	1.01 [0.96, 1.06]	0.752	1.03 [0.98, 1.08]	0.195	0.98 [0.94, 1.03]	0.518	0.97 [0.91, 1.02]	0.263	0.95 [0.91, 1.00]	0.043
Cannabis × Year	1.05 [0.95, 1.17]	0.361	1.03 [0.93, 1.15]	0.587	1.05 [0.94, 1.16]	0.376	1.05 [0.94, 1.17]	0.378	1.06 [0.95, 1.17]	0.304
IM × Cannabis × Year	1.05 [0.94, 1.16]	0.380	1.10 [0.98, 1.22]	0.100	0.97 [0.85, 1.11]	0.670	1.20 [1.07, 1.34]	0.001	1.03 [0.94, 1.13]	0.518

*IM* respective immune marker in each model, see column heading, *OR* Odds ratio, *CI* 95% confidence interval [lower and upper boundaries]. All effects are controlled for baseline variables age, sex, daily nicotine consumption and antipsychotic medication (PDD/DDD). Their respective effects (i.e., full model results) are provided in the Supplementary Information (Table S2-6).

**Fig. 1:**
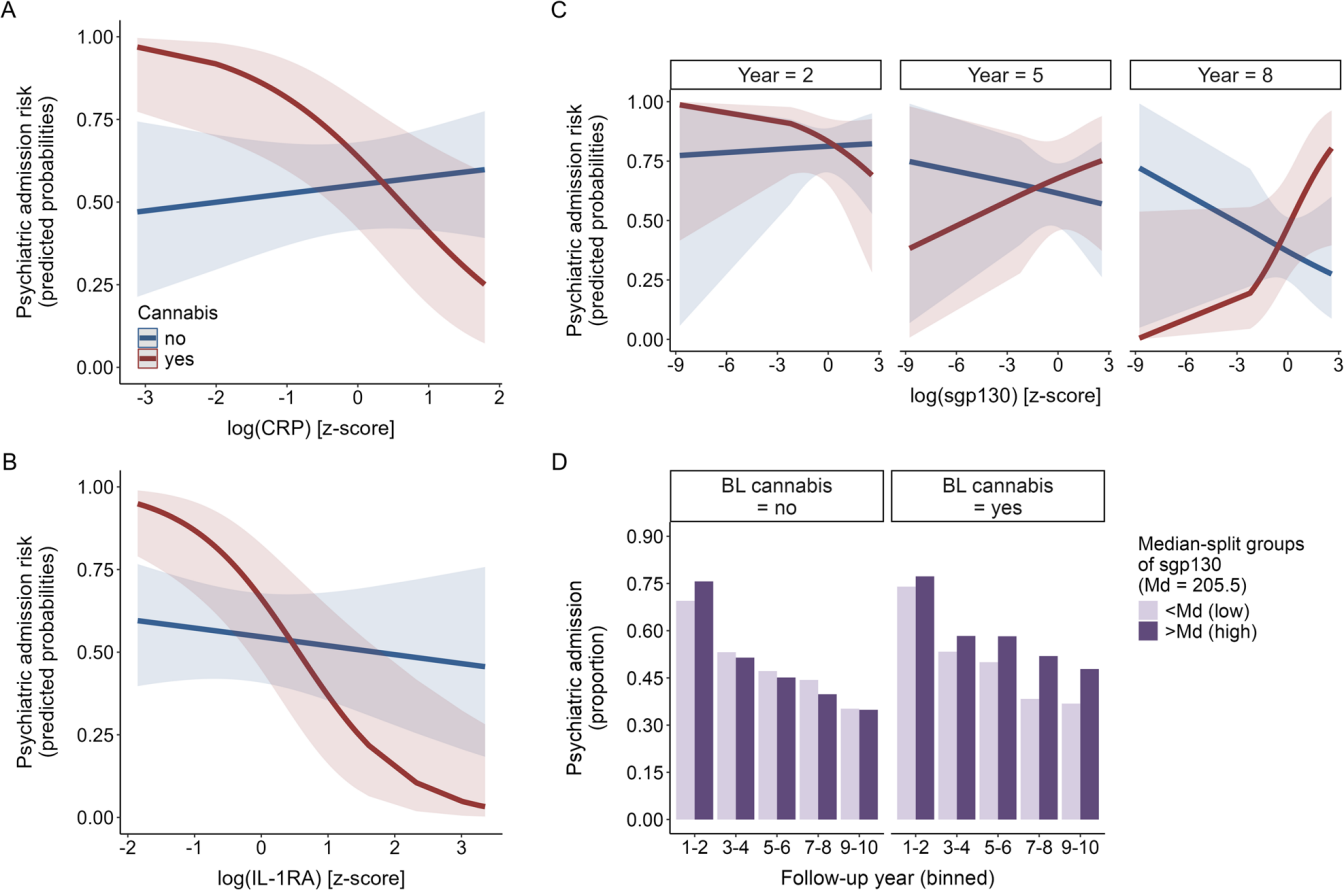
Psychiatric admission risk: significant immune marker × cannabis (× year) interactions. **A**–**C** Predicted probabilities of the significant interaction terms from the logistic mixed effects models, with CRP*cannabis use (**A**), IL-1RA*cannabis use (**B**), and sgp130*cannabis use*year (**C**). Colors indicate cannabis use category; shaded areas are 95% confidence intervals. For demonstration purposes, only 3 of the 10 follow-up years are displayed in **C**, see Figure S2 for all. **D** Summarized raw data, for demonstration purposes only: proportion of participants with psychiatric admissions per binned follow-up year pair, plotted separately for cannabis users and non-users, colored by level of sgp130 baseline concentration (above or below the median of the sample with available data on psychiatric readmissions, sgp130 levels, and cannabis use).

Specifically, the effect of both CRP (OR = 0.27, *p* = 0.009, CI [0.1, 0.72]) and IL-1RA (OR = 0.20, *p* = 0.002, CI [0.07, 0.55]) on psychiatric admission risk varied by cannabis use, with a negative association observed in users but not in non-users (Fig. 1A, B). That is, in cannabis-using individuals, high baseline levels of these immune markers were associated with lower psychiatric admission risk over the 10-year follow-up period. In contrast, no association was observed in non-users (see Supplementary Tables S17, 18 for post-hoc analyses stratified by cannabis use).

The effect of sgp130 was also dependent on cannabis use, but this varied additionally by follow-up year (OR = 1.20, *p* = 0.001, CI [1.07, 1.34]). With increasing years since baseline, higher sgp130 levels were associated with higher admission risk in cannabis users (Fig. 1C, D and S2; see Supplementary Table S19 for post-hoc analyses stratified by cannabis use).

**Fig. 2:**
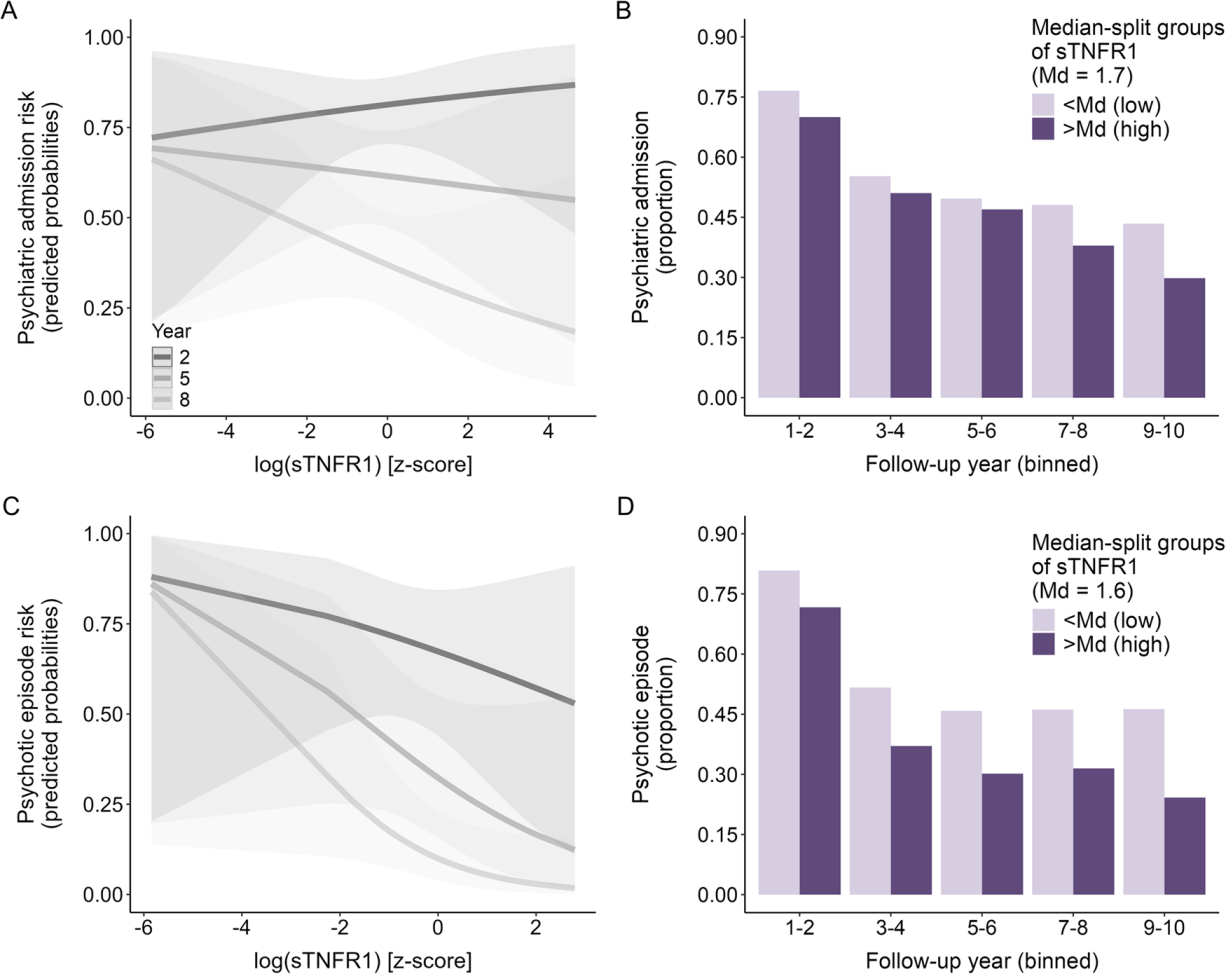
Psychiatric admission and psychotic episode risk: significant immune marker × year interaction. **A** and **C** Predicted probabilities of the significant sTNFR1*year interaction term from the logistic mixed effects models with either psychiatric admission (**A**) or psychotic episode (**C**) as outcome. Colors indicate follow-up year; shaded areas are 95% confidence intervals. For demonstration purposes, only 3 of the 10 follow-up years are displayed. **B** and **D** Summarized raw data, for demonstration purposes only: proportion of participants with psychiatric admissions (**B**) or psychotic episodes (**D**) per binned follow-up year pair, colored by level of sTNFR1 baseline concentration (above or below the median of the sample with available data on the respective outcome, and sTNFR1 levels).

Lastly, and independently of cannabis use, the effect of sTNFR1 on admission risk varied by follow-up year, albeit with a small effect size (OR = 0.95, *p* = 0.043, CI [0.91, 1.00]). Here, higher baseline concentrations of sTNFR1 were increasingly associated with a lower risk of psychiatric admission as time progressed (Fig. 2A, B).

### Interview-based analyses

To assess whether observations from the registry-based analyses could be replicated when investigating positive psychotic episodes specifically, and whether they would extend to changes in positive symptom severity, additional analyses were conducted, using the subsample of FEP participants with available clinical interview data at the 10-year follow-up (*n* = 132).

#### Psychotic episodes during follow-up

Like the registry-based analyses, logistic mixed effects models with psychotic episodes per follow-up year as the dependent variable revealed no significant main effects of either immune marker levels or cannabis use (Table 3). Instead, significant interactions between some of the immune markers, cannabis use and/or year were observed, although fewer than in registry-based analyses.

**Table 3:** Main and interaction effects for each immune marker model predicting psychotic episodes or change in positive psychotic symptom severity (interview-based analyses).

	CRP		IL-1RA		sIL-2R		sgp130		sTNFR1	
*Model (outcome): psychotic episode risk*										
*predictor*	*OR [CI]*	*p*	*OR [CI]*	*p*	*OR [CI]*	*p*	*OR [CI]*	*p*	*OR [CI]*	*p*
IM	1.03 [0.46, 2.31]	0.952	1.45 [0.70, 3.02]	0.320	1.21 [0.59, 2.49]	0.603	1.07 [0.45, 2.51]	0.885	0.93 [0.48, 1.82]	0.837
Cannabis	2.28 [0.41, 12.67]	0.348	2.02 [0.36, 11.48]	0.428	1.58 [0.27, 9.29]	0.613	2.52 [0.41, 15.41]	0.317	2.21 [0.40, 12.31]	0.366
Year	0.62 [0.57, 0.68]	<0.001	0.62 [0.56, 0.68]	<0.001	0.62 [0.57, 0.68]	<0.001	0.62 [0.57, 0.68]	<0.001	0.61 [0.56, 0.68]	<0.001
IM × Cannabis	0.91 [0.17, 4.77]	0.908	0.29 [0.07, 1.31]	0.109	0.97 [0.01, 76.77]	0.989	0.30 [0.05, 1.69]	0.172	0.22 [0.03, 1.42]	0.111
IM × Year	0.99 [0.90, 1.09]	0.857	0.94 [0.87, 1.02]	0.167	1.00 [0.92, 1.09]	0.985	0.95 [0.86, 1.05]	0.358	0.93 [0.87, 1.00]	0.038
Cannabis × Year	1.06 [0.88, 1.29]	0.525	1.06 [0.88, 1.30]	0.530	1.08 [0.89, 1.32]	0.421	1.02 [0.82, 1.26]	0.874	1.11 [0.92, 1.35]	0.264
IM × Cannabis × Year	0.88 [0.72, 1.07]	0.192	1.20 [1.02, 1.40]	0.024	0.85 [0.52, 1.38]	0.511	1.23 [1.00, 1.52]	0.056	1.08 [0.88, 1.32]	0.475
***Model (outcome): positive symptom change (ΔPANSS-Pos)***										
***predictor***	***b [CI]***	***p***	***b [CI]***	***p***	***b [CI]***	***p***	***b [CI]***	***p***	***b [CI]***	***p***
IM	−0.05 [−0.14, 0.05]	0.349	0.00 [−0.10, 0.09]	0.943	−0.06 [−0.15, 0.03]	0.169	0.04 [−0.07, 0.14]	0.469	0.00 [−0.10, 0.10]	0.966
Cannabis	−0.20 [−0.40, 0.01]	0.056	−0.16 [−0.37, 0.04]	0.120	−0.18 [−0.40, 0.04]	0.114	−0.15 [−0.35, 0.06]	0.160	−0.18 [−0.39, 0.03]	0.088
IM × Cannabis	0.13 [−0.07, 0.33]	0.186	−0.11 [−0.30, 0.09]	0.274	0.15 [−0.40, 0.69]	0.596	−0.21 [−0.41, −0.02]	0.034	−0.04 [−0.26, 0.19]	0.749

*IM* respective immune marker in each model, see column heading, *OR* Odds ratio, *b* regression coefficient, *CI* 95% confidence interval [lower and upper boundaries]. All effects are controlled for baseline variables age, sex, daily nicotine consumption and antipsychotic medication (PDD/DDD), and (in PANSS model) baseline positive symptom scores (PANSS-Pos). Their respective effects (i.e., full model results) are provided in the Supplementary Information (Table S7–16).

**Fig. 3:**
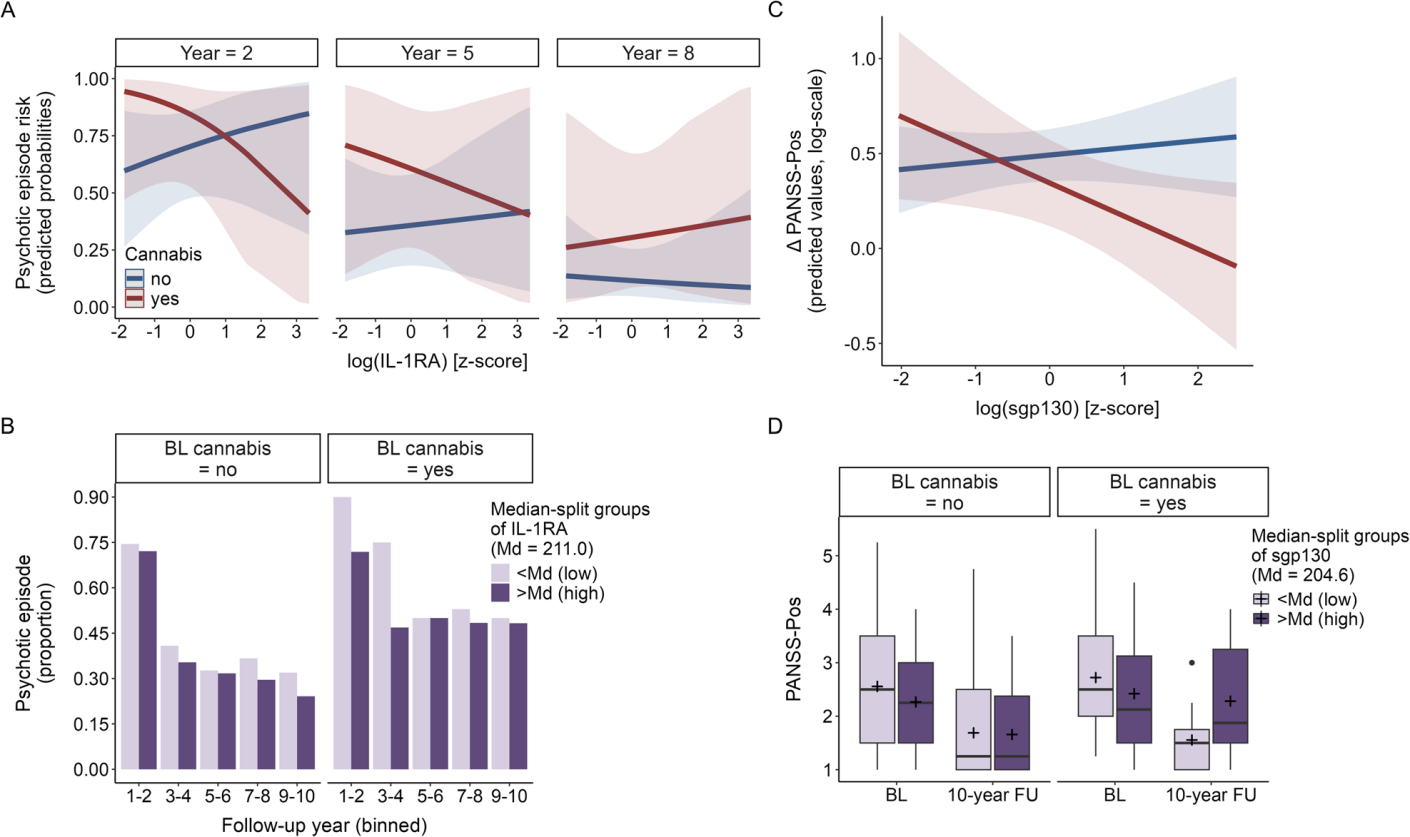
Psychotic episode risk and change in positive psychotic symptom severity (interview-based analyses): significant immune marker × cannabis (× year) interactions. **A** *Psychotic episode risk*: predicted probabilities of the significant interaction term from the logistic mixed effects model: IL-1RA*cannabis use*year. Colors indicate cannabis use category; shaded areas are 95% confidence intervals. For demonstration purposes, only 3 of the 10 follow-up years are displayed, see Figure S3 for all. **B** Summarized raw data, for demonstration purposes only: proportion of participants with psychotic episodes per binned follow-up year pair, plotted separately for cannabis users and non-users, colored by level of IL-1RA baseline concentration (above or below the median of the sample with available data on psychotic episodes, IL-1RA levels, and cannabis use). **C** *ΔPANSS-Pos*: predicted values of the significant interaction term from the linear regression model: sgp130*cannabis use. Colors indicate cannabis use category; shaded areas are 95% confidence intervals. **D** Summarized raw data, for demonstration purposes only: PANSS-Pos scores at baseline and 10-year follow-up, plotted separately for cannabis users and non-users, colored by level of sgp130 baseline concentration (above or below the median of the sample with available data on PANSS-pos scores, sgp130 levels, and cannabis use).

Here, the effect of IL-1RA was dependent on cannabis use, and this varied additionally by follow-up year (OR = 1.20, *p* = 0.024, CI [1.02, 1.40]). That is, similar to registry-based analyses, higher baseline concentrations of IL-1RA were associated with a lower risk of experiencing a psychotic episode but only in cannabis-using individuals. However, this effect diminished over time and was most pronounced during earlier follow-up years (Fig. 3A, B and S3; see Supplementary Table S20 for post-hoc analyses stratified by cannabis use). While association patterns for sgp130 mirrored those of registry-based analyses, with an interaction with cannabis use and follow-up year of similar effect size, indicating an increasingly positive association in later years in cannabis users, these findings did not reach the level of significance in this smaller sample (*p* = 0.056, Table 3).

Similar to registry-based analyses, the effect of sTNFR1 on psychotic episode risk varied by follow-up year only (OR = 0.93, *p* = 0.038, CI [0.87, 1.00]). Again, higher baseline concentrations of sTNFR1 were increasingly associated with a lower risk over time and thus most strongly linked to a lower risk in later follow-up years (Fig. 2C, D).

#### Change in positive psychotic symptom severity

In linear regression analyses with ΔPANSS-Pos as the dependent variable, the effect of sgp130 was again dependent on cannabis use, while none of the other markers showed significant associations (Table 3). Specifically, in cannabis users, higher baseline concentrations of sgp130 were associated with less improvement in positive symptom severity (b = −0.21, *p* = 0.034, CI [−0.41, −0.02]; Fig. 3C, D; see Supplementary Table S21 for post-hoc analyses stratified by cannabis use).

## Discussion

This study aimed to investigate the predictive value of various immune markers for the long-term course of psychotic symptoms and relapse in FEP while considering cannabis use as a potential confounder and moderator. Apart from sTNFR1, none of the immune markers were predictive of outcomes per se and independently of cannabis use. Instead, associations between psychosis outcomes and baseline levels of CRP, IL-1RA, and sgp130 varied significantly by cannabis use status.

### Associations between immune markers and outcomes: cannabis-use independent effects

The only immune marker with a statistical effect on psychosis outcomes not moderated by cannabis use was sTNFR1, with higher baseline levels linked to lower risk of psychiatric admissions and psychotic episodes, particularly in later follow-up years. This conflicts with previous findings of primarily cross-sectional studies where higher sTNFR1 levels were linked to prior hospitalizations, treatment resistance, and poor functioning (e.g., [26, 60, 62, 63]), or where no associations between sTNFR1 levels and hospitalizations or symptom severity were observed (e.g., [90, 91]).

None of these studies, however, have considered long-term outcomes. Further, most of these studies assessed multi-episode and not FEP samples [26, 60, 62, 63] and treatment resistance was not specifically operationalized based on positive psychotic symptoms [26, 62, 63]. Importantly, concentrations of sTNFR1 have been found to increase with antipsychotic dose [63], and in response to clozapine [92]. Thus, previously observed links between levels of sTNFR1 and indicators of treatment resistance may at least partly be explained by the effects of clozapine and high doses of antipsychotics commonly administered in treatment-resistant patients. The findings of the current study may in part be affected by the relatively high percentage of individuals with a first psychotic bipolar disorder in the sample, shown to have particularly high sTNFR1 levels compared to other severe mental disorders [93] and a lower long-term risk of psychotic episode reoccurrence.

Notably, the sTNFR1 findings remain the exception in terms of cannabis-use-independent effects on psychosis outcomes. The prominent lack of main effects in covariate- and cannabis-controlled analyses and the absence of associations between most immune markers and outcomes in non-users of cannabis, challenge the assumption of a causal link between increased inflammation/immune system activation and the course of psychosis, at least as reflected by circulating levels of markers in our study. Such findings are in line with the null findings of some of the few existing longitudinal studies conducted to test the predictive value of baseline immune markers for psychosis outcomes similar to those of the current study, both in FEP [35–37] and in schizophrenia samples [31].

This does not preclude the possibility of inflammation contributing to the risk of developing a psychotic disorder initially, but might suggest that within individuals with psychosis, inflammation plays less of a causal role for positive psychotic symptom development in the long run.

### Associations between immune markers and outcomes: cannabis-use dependent effects

In cannabis users only, baseline levels of CRP and IL-1RA showed a negative association with psychiatric admission risk over 10 years, with higher levels predicting lower risk. In contrast, levels of sgp130 were positively associated with admission risk in cannabis users, though predominantly in later follow-up years. Except for the CRP findings, these observations were largely replicated in analyses using the risk of new psychotic episodes as the outcome, though here the effect of IL-1RA diminished over time, and the effect of sgp130 did not reach statistical significance. Analyses of positive psychotic symptom change from baseline to 10-year follow-up revealed a comparable and significant effect of baseline sgp130, with higher levels predicting less symptom improvement.

The apparent diverging effects of the anti-inflammatory markers IL-1RA and sgp130 may be because the release of IL-1RA, similar to CRP, is stimulated by pro-inflammatory signals, including the pro-inflammatory cytokine IL-6 [76, 94], while levels of sgp130 do not seem to correlate consistently with levels of IL-6 [80–82, 95, 96]. Hence, one might speculate that levels of CRP and IL-1RA reflect pro-inflammatory activity, and levels of sgp130 anti-inflammatory activity, suggesting that in cannabis users, higher levels of markers indicating inflammation are associated with a lower risk of adverse outcomes, and higher levels of anti-inflammatory markers with a higher risk.

These findings are in line with the cross-sectional observations by Gibson et al. [54], who found a negative association between levels of IL-6 and positive symptom severity in cannabis users, but no association in non-users. Similarly, Romeo et al. [51] found that cannabis use cessation led to an increase in CRP levels accompanied by a decrease in clinical symptom severity in individuals with schizophrenia. Not considering cannabis use in analyses but including a substantial proportion of cannabis users in their sample of individuals with psychotic disorders, Stojanovic et al. [27] also observed a negative correlation between IL-6 levels and positive symptom severity.

Again, such findings are not easily reconcilable with the assumption of a causal link between increased inflammation/immune system activation and psychotic symptoms. While the underlying mechanisms are unknown, differences in use patterns within the group of cannabis users may offer an explanation. Dose-dependent effects of cannabinoids have been observed both for inflammation and psychotic relapse risk, with more frequent use and/or use of more potent substances linked to lower levels of pro-inflammatory and higher levels of anti-inflammatory immune markers (e.g., [97–99]), while increasing psychotic relapse risk (e.g., [2, 38, 100]). Hence, while speculative, within the group of cannabis users, those with particularly low baseline levels of pro- and high levels of anti-inflammatory markers might be those who tend to use cannabis more often or in stronger concentrations and are perhaps more likely to continue using throughout the course of illness, thus suppressing inflammation but increasing relapse risk in the long run.

The early prominence of the interaction effect between IL1-RA and cannabis use on psychotic episode risk may reflect a diminishing predictive power of certain baseline characteristics as the illness progresses, with cannabis use patterns, inflammatory status, and other psychotic relapse risk factors likely changing over time. In contrast, it is unclear why baseline sgp130 in cannabis users seems more predictive of long- than short-term outcomes. However, IL-6 signaling is complex and can be both pro- and anti-inflammatory depending on context [101]. Furthermore, while sgp130 has been shown to specifically attenuate IL-6 trans-signaling, reflecting an anti-inflammatory effect, sgp130 may also influence classical IL-6 signaling depending on molar excess of soluble IL-6 receptor sIL-6R [102]. Though speculative, high baseline sgp130 may confer short-term anti-inflammatory effects but could also reflect sustained IL-6 signaling which could be maladaptive and non-beneficial in the long-term. Notably, similar nonlinear associations have been reported in patients with acute coronary syndromes, where sgp130 may reflect a shift from compensatory to detrimental gp130-mediated signaling in the presence of high levels [103].

### Limitations

Though longitudinal in nature, this study was purely observational, limiting the extent to which conclusions can be drawn about causality. The study focused on the predictive and clinically practical value of immune marker measurements, thus, only baseline levels of immune markers were considered. While most blood samples were taken close to the clinical baseline assessments (3 weeks), there was some variation among participants. Ideally, measures for all participants would be taken on the day of the assessments to minimize differences in treatment exposure. To shed more light on questions about causality and obtain a more fine-grained picture of how acute changes in immune marker levels are associated with acute changes in illness expression, future studies should measure immune markers repeatedly throughout the course of illness. Capturing time windows both during stable periods, acute relapse, and leading up to a relapse could elucidate whether increased immune marker levels precede or follow symptom exacerbation. Practically, this is difficult, however, and studies would need to be conducted at large scale so that enough sampling happens to fall into periods just before a relapse. It would further be interesting to assess immune marker levels prior to and after cannabis consumption to assess its acute and direct effects, but this remains ethically challenging.

Cannabis use was assessed in a dichotomous manner and only covering the past 6 months prior to baseline. Baseline cannabis use status was primarily considered as a confounder and/or moderator of the association between baseline immune markers and psychosis outcomes, and the study did not aim to assess effects of cannabis use on immune markers or psychosis outcomes per se. Hence, the question of potential frequency- or dose-dependent effects was less central, and the selected measure was deemed adequate for the purpose of the current study. Nevertheless, explorative subgroup analyses with information about cannabis use history, use frequency, use dosage, modes of use, and phytocannabinoid concentrations in the consumed product, would have been interesting and could have supported interpretations of observed effects. However, data at hand were insufficient for such analyses.

Lastly, although multiple potential confounders were considered in analyses, medication other than antipsychotics was not controlled for and residual confounding cannot be ruled out. The same is true for use of other recreational drugs, including alcohol. Previous research has specifically emphasized the role of cannabis both for psychosis outcomes and for immunoactivity, thus making it the main factor of interest for the current study. However, to what extent effects of other drugs, with e.g. primarily pro-inflammatory actions such as alcohol, also may moderate the relationship between immune markers and psychosis outcomes, remains to be investigated.

### Concluding remarks

The findings of the current study provide only weak support for a general predictive value of various immune markers for the long-term course of psychotic symptoms but highlight the influence of cannabis use on this relationship. In light of the known interplay between the immune and the endocannabinoid system and the high prevalence of cannabis use in psychotic disorders, the number of studies investigating the effect of cannabis use on immune markers in individuals with psychosis is strikingly low [19, 49–53]. Additional research, both of cross-sectional and longitudinal nature, is urgently needed in this area.

The findings further cast doubt on the idea that increased inflammation per se is causally linked to a more severe long-term course of psychotic symptoms. Within cannabis users, mainly reverse associations were observed, with lower inflammation indicating a higher risk for selected adverse psychosis outcomes. Such observations might suggest that cannabis use-related dysregulation in immune responses, and not increased inflammation per se, could carry a certain risk for psychotic relapse. However, they may also be attributable to unmeasured factors such as particular use patterns, and the observed associations may not be causal. Observations of association patterns in non-users also mostly suggest an absence of any associations between immune markers and psychosis outcomes. It is possible that such associations emerge primarily over shorter timespans, e.g. leading up to a psychotic relapse, or that they are more prevalent in specific subgroups.

## Supplementary information

Supplementary Information

## Data Availability

Due to ethical restrictions, the data used in the current study are not publicly available. Interested parties may request access from the corresponding author through a reasonable inquiry, subject to approval by the Regional Ethics Committee.
