# Supplementary material for: Recent cannabis use affects the association between baseline immune markers and long-term outcomes in psychosis
Source: Transl Psychiatry. 2025 Aug 14;15:282. doi: 10.1038/s41398-025-03498-x (PMC12354687; doi:10.1038/s41398-025-03498-x)
Supplement: Supplementary file 1 — Supplementary Information [file 41398_2025_3498_MOESM1_ESM.pdf]

Supplementary Information for:

Recent cannabis use affects the association between baseline immune markers and long-term outcomes in psychosis

Isabel Kreis[1,2], Kristin Fjelnseth Wold[2,3], Gina Åsbø[2,4], Camilla Bärthel Flaaten[2], Magnus Johan Engen[5], Siv Hege Lyngstad[5], Line Hustad Widing[2,6], Mashhood Ahmed Sheikh[7], Maren Caroline Frogner Werner[8], Eivind Bakken[9], Thor Ueland[7,3,10], Nils Eiel Steen[2,1,11], Ingrid Melle[2,12]

[1] Centre for Precision Psychiatry, Institute of Clinical Medicine, Faculty of Medicine, University of Oslo, Oslo, Norway

[2] Section for Clinical Psychosis Research, Department of Research and Innovation, Division of Mental Health and Addiction, Oslo University Hospital, Oslo, Norway

[3] Institute of Clinical Medicine, University of Oslo, Oslo, Norway

[4] Department of Psychology, Faculty of Social Sciences, University of Oslo, Oslo, Norway

[5] Nydalen District Psychiatric Center, Division of Mental Health and Addiction, Oslo University Hospital, Oslo, Norway

[6] Department of Child and Adolescent Psychiatry, Division of Mental Health and Substance Use, Diakonhjemmet Hospital, Oslo, Norway

[7] Research Institute of Internal Medicine, Oslo University Hospital, Rikshospitalet, Oslo

[8] Department of Research and Innovation, Clinical Neuroscience, Oslo University Hospital, Oslo, Norway

[9] Section for Precision Psychiatry, Department of Research and Innovation, Division of Mental Health and Addiction, Oslo University Hospital, Oslo, Norway

[10] Thrombosis Research Center (TREC), Division of Internal Medicine, University Hospital of North Norway, Tromsø, Norway

[11] Department of Psychiatric Research, Division of Mental Health and Substance abuse, Diakonhjemmet Hospital, Oslo, Norway

[12] Adult Psychiatry Department, Institute of Clinical Medicine, Faculty of Medicine, University of Oslo, Oslo, Norway.

Sample selection

Figure S1

Flow-chart of participation and data selection

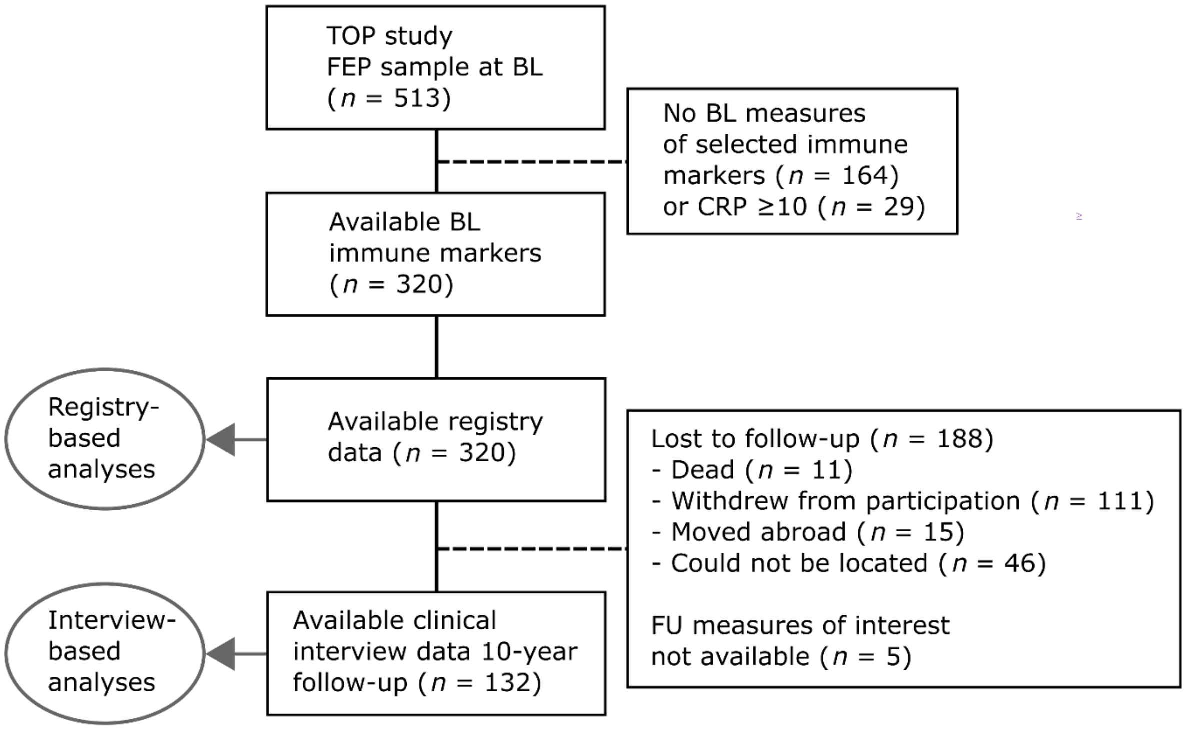

Note. BL = baseline, FU = 10-year follow-up. FU measures of interest were psychotic episodes during follow-up and change in positive psychotic symptoms from baseline to follow-up (see Clinical assessments, main text).

Supplementary table: immune markers by cannabis use status

Table S1. Immune markers by cannabis use status

**IM**	**Cannabis = No (N=242)**		**Cannabis = Yes (N=71)**		**Statistical test**	
					**Welch's *t*-test**	
	M	***SD***	***M***	***SD***	***t***	***p***
log(CRP)	-0.02	1.01	0.04	0.98	-0.44	.664
log(IL-1RA)	-0.01	1.02	0.08	0.94	-0.70	.485
log(sIL-2R)	0.02	1.09	-0.05	0.68	0.62	.536
log(sgp130)	0.01	0.87	-0.05	1.38	0.31	.760
log(sTNFR1)	-0.04	0.96	0.13	1.12	-1.15	.252
					**Mann–Whitney *U* test**	
	Md	***IQR***	***Md***	***IQR***	***U***	***p***
CRP	1.81	2.90	1.90	2.75	8432	.813
IL-1RA	192.00	281.25	281.75	325.13	7588	.238
sIL-2R	0.25	0.16	0.30	0.20	7440	.160
sgp130	203.68	52.95	210.15	64.19	7926	.505
sTNFR1	1.65	0.57	1.76	0.71	7227	.092

Note. Parametric and non-parametric statistics are provided for variables on log-scale and original scale, respectively. IM = Immune marker M = mean, SD = standard deviation, Md = median, IQR = interquartile range. Total N = 313. Missing for each variable: Cannabis = No: IL-1RA = 3, sIL-2R = 3, sgp130 = 3, sTNFR1 = 4. Cannabis = Yes: IL-1RA = 1, sIL-2R = 1, sgp130 = 1, sTNFR1 = 1.

Note that a previous study [1] contrasted some of the immune markers selected here (IL-1RA, sgp130, sTNFR1) by recent cannabis use status in a partially overlapping sample (not selected for first-episode psychosis and with different diagnosis selection criteria).

Supplementary tables: complete model results

Tables presenting the complete results of all models, including covariates.

Registry-based analyses

Table S2. Logistic mixed-effects model results with immune marker CRP (full model with covariates). Outcome = psychiatric (re)admission

**predictor**	**OR**	**CI (low)**	**CI (high)**	***p***
(Intercept)	7.01	2.13	23.00	.001
Age	0.97	0.94	1.01	.165
Sex	1.23	0.66	2.28	.515
Daily nicotine	0.52	0.27	0.98	.044
PDD/DDD	2.94	1.89	4.58	<.001
CRP	1.06	0.68	1.65	.787
Cannabis	1.08	0.40	2.90	.881
Year	0.72	0.68	0.75	<.001
CRP*Cannabis	0.27	0.10	0.72	.009
CRP*Year	1.01	0.96	1.06	.752
Cannabis*Year	1.05	0.95	1.17	.361
CRP*Cannabis*Year	1.05	0.94	1.16	.380

Note. Fitted values: N = 312 participants (complete data on all predictors and covariates), total (repeated) observations = 2827. CRP = log- and z-transformed values of CRP levels. PDD/DDD = antipsychotic medication load. Contrasts of categorical predictors are male vs. female (sex), and yes vs. no (daily nicotine, cannabis).

Table S3. Logistic mixed-effects model results with immune marker IL-1RA (full model with covariates). Outcome = psychiatric (re)admission

**predictor**	**OR**	**CI (low)**	**CI (high)**	***p***
(Intercept)	5.46	1.67	17.78	.005
Age	0.98	0.95	1.02	.366
Sex	1.33	0.71	2.49	.367
Daily nicotine	0.49	0.26	0.94	.033
PDD/DDD	2.83	1.82	4.41	<.001
IL-1RA	0.75	0.48	1.17	.201
Cannabis	1.38	0.50	3.79	.534
Year	0.72	0.68	0.76	<.001
IL-1RA*Cannabis	0.20	0.07	0.55	.002
IL-1RA*Year	1.03	0.98	1.08	.195
Cannabis*Year	1.03	0.93	1.15	.587
IL-1RA*Cannabis*Year	1.10	0.98	1.22	.100

Note. Fitted values: N = 308 participants (complete data on all predictors and covariates), total (repeated) observations = 2791. IL-1RA = log- and z-transformed values of IL-1RA levels. PDD/DDD = antipsychotic medication load. Contrasts of categorical predictors are male vs. female (sex), and yes vs. no (daily nicotine, cannabis).

Table S4. Logistic mixed-effects model results with immune marker sIL-2R (full model with covariates). Outcome = psychiatric (re)admission

**predictor**	**OR**	**CI (low)**	**CI (high)**	***p***
(Intercept)	7.00	2.14	22.90	.001
Age	0.98	0.94	1.01	.234
Sex	1.22	0.64	2.30	.548
Daily nicotine	0.47	0.25	0.91	.024
PDD/DDD	2.73	1.75	4.26	<.001
sIL-2R	1.11	0.73	1.70	.622
Cannabis	1.08	0.40	2.90	.884
Year	0.72	0.68	0.76	<.001
sIL-2R*Cannabis	0.70	0.20	2.46	.573
sIL-2R*Year	0.98	0.94	1.03	.518
Cannabis*Year	1.05	0.94	1.16	.376
sIL-2R*Cannabis*Year	0.97	0.85	1.11	.670

Note. Fitted values: N = 308 participants (complete data on all predictors and covariates), total (repeated) observations = 2791. sIL-2R = log- and z-transformed values of sIL-2R levels. PDD/DDD = antipsychotic medication load. Contrasts of categorical predictors are male vs. female (sex), and yes vs. no (daily nicotine, cannabis).

Table S5. Logistic mixed-effects model results with immune marker sgp130 (full model with covariates). Outcome = psychiatric (re)admission

**predictor**	**OR**	**CI (low)**	**CI (high)**	***p***
(Intercept)	6.87	2.04	23.13	.002
Age	0.98	0.94	1.02	.263
Sex	1.31	0.66	2.58	.436
Daily nicotine	0.46	0.24	0.90	.024
PDD/DDD	2.66	1.68	4.20	<.001
sgp130	1.10	0.64	1.87	.734
Cannabis	1.04	0.38	2.85	.938
Year	0.72	0.68	0.75	<.001
sgp130*Cannabis	0.50	0.22	1.12	.091
sgp130*Year	0.97	0.91	1.02	.263
Cannabis*Year	1.05	0.94	1.17	.378
sgp130*Cannabis*Year	1.20	1.07	1.34	.001

Note. Fitted values: N = 308 participants (complete data on all predictors and covariates), total (repeated) observations = 2791. sgp130 = log- and z-transformed values of sgp130 levels. PDD/DDD = antipsychotic medication load. Contrasts of categorical predictors are male vs. female (sex), and yes vs. no (daily nicotine, cannabis).

Table S6. Logistic mixed-effects model results with immune marker sTNFR1 (full model with covariates). Outcome = psychiatric (re)admission

**predictor**	**OR**	**CI (low)**	**CI (high)**	***p***
(Intercept)	6.22	1.85	20.90	.003
Age	0.98	0.95	1.02	.338
Sex	1.24	0.65	2.33	.514
Daily nicotine	0.50	0.26	0.96	.036
PDD/DDD	2.78	1.78	4.34	<.001
sTNFR1	1.21	0.77	1.90	.418
Cannabis	1.08	0.40	2.90	.883
Year	0.72	0.68	0.75	<.001
sTNFR1*Cannabis	0.65	0.27	1.53	.318
sTNFR1*Year	0.95	0.91	1.00	.043
Cannabis*Year	1.06	0.95	1.17	.304
sTNFR1*Cannabis*Year	1.03	0.94	1.13	.518

Note. Fitted values: N = 307 participants (complete data on all predictors and covariates), total (repeated) observations = 2781. sTNFR1 = log- and z-transformed values of sTNFR1 levels. PDD/DDD = antipsychotic medication load. Contrasts of categorical predictors are male vs. female (sex), and yes vs. no (daily nicotine, cannabis).

Interview-based analyses: psychotic episodes during follow-up

Table S7. Logistic mixed-effects model results with immune marker CRP (full model with covariates). Outcome = psychotic episode

**predictor**	**OR**	**CI (low)**	**CI (high)**	***p***
(Intercept)	3.02	0.36	25.67	.311
Age	1.00	0.93	1.07	.947
Sex	2.16	0.72	6.47	.167
Daily nicotine	1.53	0.49	4.75	.465
PDD/DDD	1.92	0.79	4.63	.149
CRP	1.03	0.46	2.31	.952
Cannabis	2.28	0.41	12.67	.348
Year	0.62	0.57	0.68	<.001
CRP*Cannabis	0.91	0.17	4.77	.908
CRP*Year	0.99	0.90	1.09	.857
Cannabis*Year	1.06	0.88	1.29	.525
CRP*Cannabis*Year	0.88	0.72	1.07	.192

Note. Fitted values: N = 123 participants (complete data on all predictors and covariates), total (repeated) observations = 1123. CRP = log- and z-transformed values of CRP levels. PDD/DDD = antipsychotic medication load. Contrasts of categorical predictors are male vs. female (sex), and yes vs. no (daily nicotine, cannabis).

Table S8. Logistic mixed-effects model results with immune marker IL-1RA (full model with covariates). Outcome = psychotic episode

**predictor**	**OR**	**CI (low)**	**CI (high)**	***p***
(Intercept)	6.05	0.74	49.20	.093
Age	0.99	0.92	1.05	.655
Sex	2.04	0.70	5.97	.194
Daily nicotine	1.23	0.40	3.78	.718
PDD/DDD	1.68	0.71	4.02	.240
IL-1RA	1.45	0.70	3.02	.320
Cannabis	2.02	0.36	11.48	.428
Year	0.62	0.56	0.68	<.001
IL-1RA*Cannabis	0.29	0.07	1.31	.109
IL-1RA*Year	0.94	0.87	1.02	.167
Cannabis*Year	1.06	0.88	1.30	.530
IL-1RA*Cannabis*Year	1.20	1.02	1.40	.024

Note. Fitted values: N = 119 participants (complete data on all predictors and covariates), total (repeated) observations = 1086. IL-1RA = log- and z-transformed values of IL-1RA levels. PDD/DDD = antipsychotic medication load. Contrasts of categorical predictors are male vs. female (sex), and yes vs. no (daily nicotine, cannabis).

Table S9. Logistic mixed-effects model results with immune marker sIL-2R (full model with covariates). Outcome = psychotic episode

**predictor**	**OR**	**CI (low)**	**CI (high)**	***p***
(Intercept)	5.77	0.73	45.76	.097
Age	0.99	0.93	1.05	.678
Sex	1.82	0.60	5.55	.290
Daily nicotine	1.30	0.43	3.93	.643
PDD/DDD	1.67	0.70	3.96	.244
sIL-2R	1.21	0.59	2.49	.603
Cannabis	1.58	0.27	9.29	.613
Year	0.62	0.57	0.68	<.001
sIL-2R*Cannabis	0.97	0.01	76.77	.989
sIL-2R*Year	1.00	0.92	1.09	.985
Cannabis*Year	1.08	0.89	1.32	.421
sIL-2R*Cannabis*Year	0.85	0.52	1.38	.511

Note. Fitted values: N = 119 participants (complete data on all predictors and covariates), total (repeated) observations = 1086. sIL-2R = log- and z-transformed values of sIL-2R levels. PDD/DDD = antipsychotic medication load. Contrasts of categorical predictors are male vs. female (sex), and yes vs. no (daily nicotine, cannabis).

Table S10. Logistic mixed-effects model results with immune marker sgp130 (full model with covariates). Outcome = psychotic episode

**predictor**	**OR**	**CI (low)**	**CI (high)**	***p***
(Intercept)	5.11	0.61	42.82	.133
Age	0.99	0.93	1.05	.704
Sex	2.12	0.68	6.60	.193
Daily nicotine	1.38	0.44	4.29	.582
PDD/DDD	1.73	0.72	4.19	.222
sgp130	1.07	0.45	2.51	.885
Cannabis	2.52	0.41	15.41	.317
Year	0.62	0.57	0.68	<.001
sgp130*Cannabis	0.30	0.05	1.69	.172
sgp130*Year	0.95	0.86	1.05	.358
Cannabis*Year	1.02	0.82	1.26	.874
sgp130*Cannabis*Year	1.23	1.00	1.52	.056

Note. Fitted values: N = 119 participants (complete data on all predictors and covariates), total (repeated) observations = 1086. sgp130 = log- and z-transformed values of sgp130 levels. PDD/DDD = antipsychotic medication load. Contrasts of categorical predictors are male vs. female (sex), and yes vs. no (daily nicotine, cannabis).

Table S11. Logistic mixed-effects model results with immune marker sTNFR1 (full model with covariates). Outcome = psychotic episode

predictor	OR	CI (low)	CI (high)	*p*
(Intercept)	4.66	0.57	38.05	.151
Age	0.99	0.93	1.06	.841
Sex	2.08	0.72	6.00	.177
Daily nicotine	1.27	0.42	3.85	.670
PDD/DDD	1.56	0.67	3.65	.305
sTNFR1	0.93	0.48	1.82	.837
Cannabis	2.21	0.40	12.31	.366
Year	0.61	0.56	0.68	<.001
sTNFR1*Cannabis	0.22	0.03	1.42	.111
sTNFR1*Year	0.93	0.87	1.00	.038
Cannabis*Year	1.11	0.92	1.35	.264
sTNFR1*Cannabis*Year	1.08	0.88	1.32	.475

Note. Fitted values: N = 118 participants (complete data on all predictors and covariates), total (repeated) observations = 1079. sTNFR1 = log- and z-transformed values of sTNFR1 levels. PDD/DDD = antipsychotic medication load. Contrasts of categorical predictors are male vs. female (sex), and yes vs. no (daily nicotine, cannabis).

Interview-based analyses: change in positive symptom severity

Table S12. Linear regression model results with immune marker CRP (full model with covariates). Outcome = positive psychotic symptom change (ΔPANSS-Pos)

**predictor**	***b***	**CI (low)**	**CI (high)**	***p***
(Intercept)	0.04	-0.30	0.38	.824
Age	-0.01	-0.02	0.00	.205
Sex	-0.13	-0.28	0.03	.118
Daily nicotine	-0.02	-0.19	0.14	.781
PDD/DDD	0.05	-0.08	0.17	.443
PANSS-Pos BL	0.70	0.54	0.87	.000
CRP	-0.05	-0.14	0.05	.349
Cannabis	-0.20	-0.40	0.01	.056
CRP*Cannabis	0.13	-0.07	0.33	.186

Note. Fitted values: N = 127 participants (complete data on all predictors and covariates). CRP = log- and z-transformed values of CRP levels. PDD/DDD = antipsychotic medication load, PANSS-Pos BL = log-transformed baseline values of the positive symptoms PANSS subscale. Contrasts of categorical predictors are male vs. female (sex), and yes vs. no (daily nicotine, cannabis).

Table S13. Linear regression model results with immune marker IL-1RA (full model with covariates). Outcome = positive psychotic symptom change (ΔPANSS-Pos)

**predictor**	***b***	**CI (low)**	**CI (high)**	***p***
(Intercept)	0.05	-0.30	0.39	.796
Age	-0.01	-0.02	0.00	.201
Sex	-0.13	-0.29	0.03	.107
Daily nicotine	-0.01	-0.18	0.16	.901
PDD/DDD	0.06	-0.07	0.19	.340
PANSS-Pos BL	0.69	0.52	0.86	.000
IL-1RA	0.00	-0.10	0.09	.943
Cannabis	-0.16	-0.37	0.04	.120
IL-1RA*Cannabis	-0.11	-0.30	0.09	.274

Note. Fitted values: N = 123 participants (complete data on all predictors and covariates). IL-1RA = log- and z-transformed values of IL-1RA levels. PDD/DDD = antipsychotic medication load, PANSS-Pos BL = log-transformed baseline values of the positive symptoms PANSS subscale. Contrasts of categorical predictors are male vs. female (sex), and yes vs. no (daily nicotine, cannabis).

Table S14. Linear regression model results with immune marker sIL-2R (full model with covariates). Outcome = positive psychotic symptom change (ΔPANSS-Pos)

**predictor**	***b***	**CI (low)**	**CI (high)**	***p***
(Intercept)	0.04	-0.30	0.39	.809
Age	-0.01	-0.02	0.00	.168
Sex	-0.10	-0.27	0.07	.250
Daily nicotine	-0.01	-0.18	0.15	.890
PDD/DDD	0.07	-0.06	0.19	.307
PANSS-Pos BL	0.68	0.51	0.85	.000
sIL-2R	-0.06	-0.15	0.03	.169
Cannabis	-0.18	-0.40	0.04	.114
sIL-2R*Cannabis	0.15	-0.40	0.69	.596

Note. Fitted values: N = 123 participants (complete data on all predictors and covariates). sIL-2R = log- and z-transformed values of sIL-2R levels. PDD/DDD = antipsychotic medication load, PANSS-Pos BL = log-transformed baseline values of the positive symptoms PANSS subscale. Contrasts of categorical predictors are male vs. female (sex), and yes vs. no (daily nicotine, cannabis).

Table S15. Linear regression model results with immune marker sgp130 (full model with covariates). Outcome = positive psychotic symptom change (ΔPANSS-Pos)

**predictor**	***b***	**CI (low)**	**CI (high)**	***p***
(Intercept)	0.07	-0.27	0.42	.666
Age	-0.01	-0.02	0.00	.138
Sex	-0.16	-0.32	0.01	.063
Daily nicotine	0.00	-0.16	0.16	.997
PDD/DDD	0.07	-0.05	0.20	.250
PANSS-Pos BL	0.69	0.52	0.85	.000
sgp130	0.04	-0.07	0.14	.469
Cannabis	-0.15	-0.35	0.06	.160
sgp130*Cannabis	-0.21	-0.41	-0.02	.034

Note. Fitted values: N = 123 participants (complete data on all predictors and covariates). sgp130 = log- and z-transformed values of sgp130 levels. PDD/DDD = antipsychotic medication load, PANSS-Pos BL = log-transformed baseline values of the positive symptoms PANSS subscale. Contrasts of categorical predictors are male vs. female (sex), and yes vs. no (daily nicotine, cannabis).

Table S16. Linear regression model results with immune marker sTNFR1 (full model with covariates). Outcome = positive psychotic symptom change (ΔPANSS-Pos)

**predictor**	***b***	**CI (low)**	**CI (high)**	***p***
(Intercept)	0.09	-0.27	0.46	.606
Age	-0.01	-0.02	0.00	.114
Sex	-0.12	-0.28	0.05	.158
Daily nicotine	-0.03	-0.20	0.14	.753
PDD/DDD	0.06	-0.07	0.19	.364
PANSS-Pos BL	0.69	0.52	0.86	.000
sTNFR1	0.00	-0.10	0.10	.966
Cannabis	-0.18	-0.39	0.03	.088
sTNFR1*Cannabis	-0.04	-0.26	0.19	.749

Note. Fitted values: N = 122 participants (complete data on all predictors and covariates). sTNFR1 = log- and z-transformed values of sTNFR1 levels. PDD/DDD = antipsychotic medication load, PANSS-Pos BL = log-transformed baseline values of the positive symptoms PANSS subscale. Contrasts of categorical predictors are male vs. female (sex), and yes vs. no (daily nicotine, cannabis).

Supplementary tables: post-hoc analyses

Analyses stratified by cannabis use status, conducted for all outcome and immune marker combinations where the interaction between immune marker levels and cannabis use reached significance. Supplementary figures are added to visualize the significant 3-way interaction effects from the original, unstratified models. Here, predicted risk based on the respective full model is displayed by plotting marginal effects using the sjPlot package (version 2.8.15)[2], with effects displayed at each level of follow-up year (1 – 10).

Registry-based analyses

Table S17. Main and interaction effects of models with immune marker CRP, stratified by cannabis use. Outcome = psychiatric (re)admission

	**Cannabis use = no**		**Cannabis use = yes**	
**predictor**	**OR [CI]**	***p***	**OR [CI]**	***p***
CRP	1.04 [0.67,1.6]	.863	0.29 [0.12,0.75]	.011
Year	0.72 [0.68,0.76]	<.001	0.75 [0.68,0.82]	<.001
CRP*Year	1.01 [0.96,1.06]	.761	1.06 [0.97,1.16]	.226

Note. All effects are controlled for baseline variables age, sex, daily nicotine consumption and antipsychotic medication (PDD/DDD).

Table S18. Main and interaction effects of models with immune marker IL-1RA, stratified by cannabis use. Outcome = psychiatric (re)admission

	**Cannabis use = no**		**Cannabis use = yes**	
**predictor**	**OR [CI]**	***p***	**OR [CI]**	***p***
IL-1RA	0.77 [0.5,1.18]	0.231	0.13 [0.05,0.38]	<.001
Year	0.72 [0.69,0.76]	<.001	0.73 [0.66,0.81]	<.001
IL-1RA*Year	1.03 [0.98,1.08]	0.196	1.14 [1.03,1.26]	.012

Note. All effects are controlled for baseline variables age, sex, daily nicotine consumption and antipsychotic medication (PDD/DDD).

Table S19. Main and interaction effects of models with immune marker sgp130, stratified by cannabis use. Outcome = psychiatric (re)admission

	**Cannabis use = no**		**Cannabis use = yes**	
**predictor**	**OR [CI]**	***p***	**OR [CI]**	***p***
sgp130	1.12 [0.67,1.87]	.677	0.54 [0.26,1.14]	.106
Year	0.72 [0.68,0.76]	<.001	0.73 [0.66,0.82]	<.001
sgp130*Year	0.97 [0.91,1.02]	.257	1.19 [1.07,1.32]	.001

Note. All effects are controlled for baseline variables age, sex, daily nicotine consumption and antipsychotic medication (PDD/DDD).

Figure S2

Psychiatric admission risk: immune marker (sgp130) × cannabis × year interaction

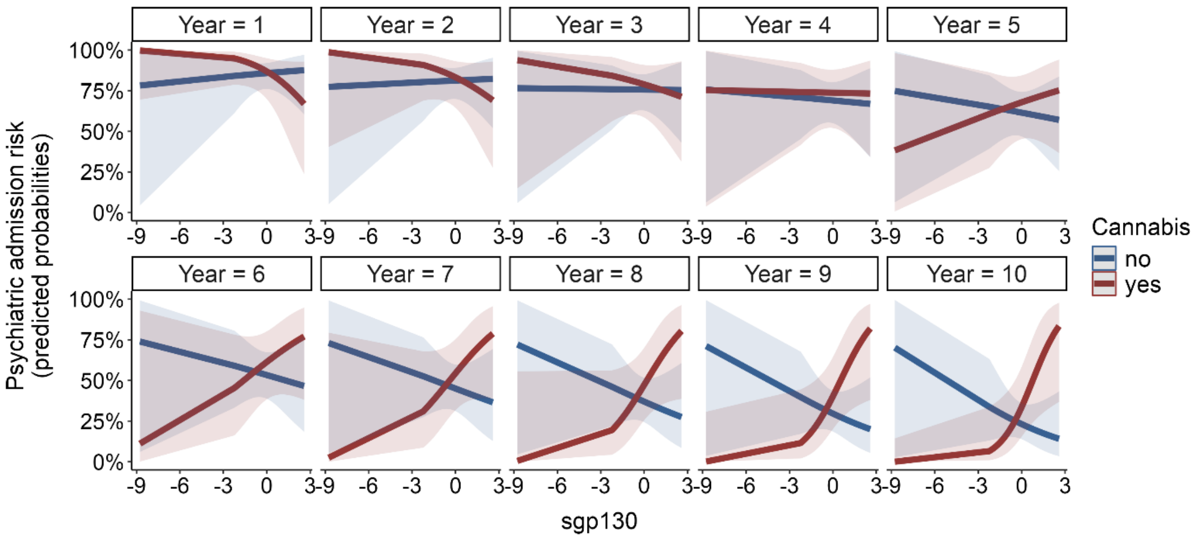

Interview-based analyses

Table S20. Main and interaction effects of models with immune marker IL-1RA, stratified by cannabis use. Outcome = psychotic episode

	Cannabis use = no		Cannabis use = yes	
**predictor**	**OR [CI]**	***p***	**OR [CI]**	***p***
IL-1RA	1.6 [0.79,3.27]	0.195	0.28 [0.08,1.02]	.054
Year	0.62 [0.56,0.68]	<.001	0.64 [0.53,0.78]	<.001
IL-1RA*Year	0.94 [0.87,1.02]	0.149	1.15 [1,1.32]	.053

Note. All effects are controlled for baseline variables age, sex, daily nicotine consumption and antipsychotic medication (PDD/DDD).

Figure S3

Psychotic episode risk: immune marker (IL-1RA) × cannabis × year interaction

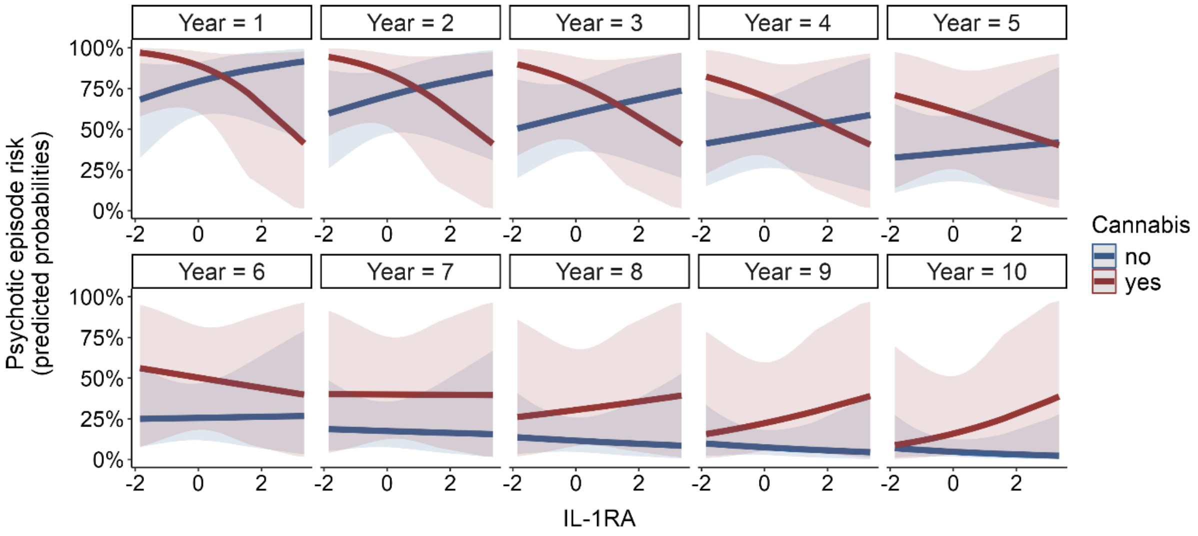

Table S21. Main effects of linear regression models with immune marker sgp130, stratified by cannabis use. Outcome = positive psychotic symptom change (ΔPANSS-Pos)

	Cannabis use = no		Cannabis use = yes	
**predictor**	***b*** **[CI]**	***p***	***b*** **[CI]**	***p***
sgp130	0.03 [-0.08, 0.13]	.624	-0.18 [-0.35, -0.01]	.035

Note. All effects are controlled for baseline variables age, sex, daily nicotine consumption, antipsychotic medication (PDD/DDD), and baseline positive symptom scores (PANSS-Pos).

References (suppl.)

1. Szabo A, Akkouh IA, Ueland T, Lagerberg TV, Dieset I, Bjella T *et al.* Cannabis Use Is Associated With Increased Levels of Soluble gp130 in Schizophrenia but Not in Bipolar Disorder. *Front Psychiatry* 2020; **11:** 642.
2. Lüdecke, D. (2024). *sjPlot: Data Visualization for Statistics in Social Science*. R package version 2.8.16, https://CRAN.R-project.org/package=sjPlot.
